# Delayed Facial Nerve Palsy Developing After Surgery for a Benign Parotid Gland Tumor

**DOI:** 10.7759/cureus.98700

**Published:** 2025-12-08

**Authors:** Kiyomi Kuba, Masaya Umino, Masami Osaki, Akio Hatanaka, Keitaro Nagano

**Affiliations:** 1 Otolaryngology - Head and Neck Surgery, Ageo Central General Hospital, Ageo, JPN

**Keywords:** benign parotid gland tumor, delayed facial nerve palsy, parotid gland surgery, salivary gland tumor, varicella-zoster virus

## Abstract

Facial nerve palsy is the most serious complication to be aware of in surgery for parotid gland tumors. It occurs due to various factors including direct intraoperative injury, traction, thermal injury, ischemia, and anatomical variations. While most cases occur immediately after surgery, some may develop within the first 24 to 72 hours due to postoperative edema or hematoma. Delayed facial nerve paralysis occurring more than 72 hours after surgery is extremely rare. We report a case of a 60-year-old woman who developed delayed facial nerve palsy 16 days after parotid gland surgery. Mild paralysis of the lower lip was observed postoperatively, but no abnormalities were detected in other facial muscles. The postoperative course was uneventful, and the patient was discharged on the eighth day. However, on the 10th day after the surgery, pain appeared around her ear, and severe facial nerve paralysis developed on the 16th day. Pain was present from the ear to the scalp of the temporal region, but no objective findings such as postoperative infection, edema, hematoma, or tumor recurrence were observed. The patient received combined therapy with steroids and antiviral agents based on the treatment for idiopathic facial nerve palsy, and the paralysis fully recovered after four months. Delayed facial nerve palsy following parotid gland tumor surgery is extremely rare, and it is important to investigate causative factors before initiating treatment.

## Introduction

In parotid gland tumor surgery, we must pay attention to complications such as facial nerve palsy, postoperative hematoma, salivary fistula, and Frey's syndrome, in addition to achieving pathological control of the tumor. The incidence of facial nerve palsy after parotidectomy is reported to be approximately 23-38% for temporary palsy and 5-13% for permanent palsy, with the permanent palsy rate being as low as 1.6% when limited to benign tumors [1]. Facial nerve palsy typically presents immediately following surgery. However, it may also manifest within the first 24-72 hours postoperatively, most commonly secondary to hematoma or edema. In contrast, delayed facial nerve palsy (DFNP) occurring more than 72 hours after surgery is exceedingly rare [2]. We encountered a case where severe facial nerve palsy appeared more than two weeks after surgery. Although delayed postoperative facial nerve palsy is predominantly reported following middle ear or temporal bone surgery, cases occurring after parotid gland tumor resection have also been documented. This report emphasizes the importance of understanding delayed paralysis following parotid surgery and the significance of early therapeutic intervention.

## Case presentation

**Figure 1:**
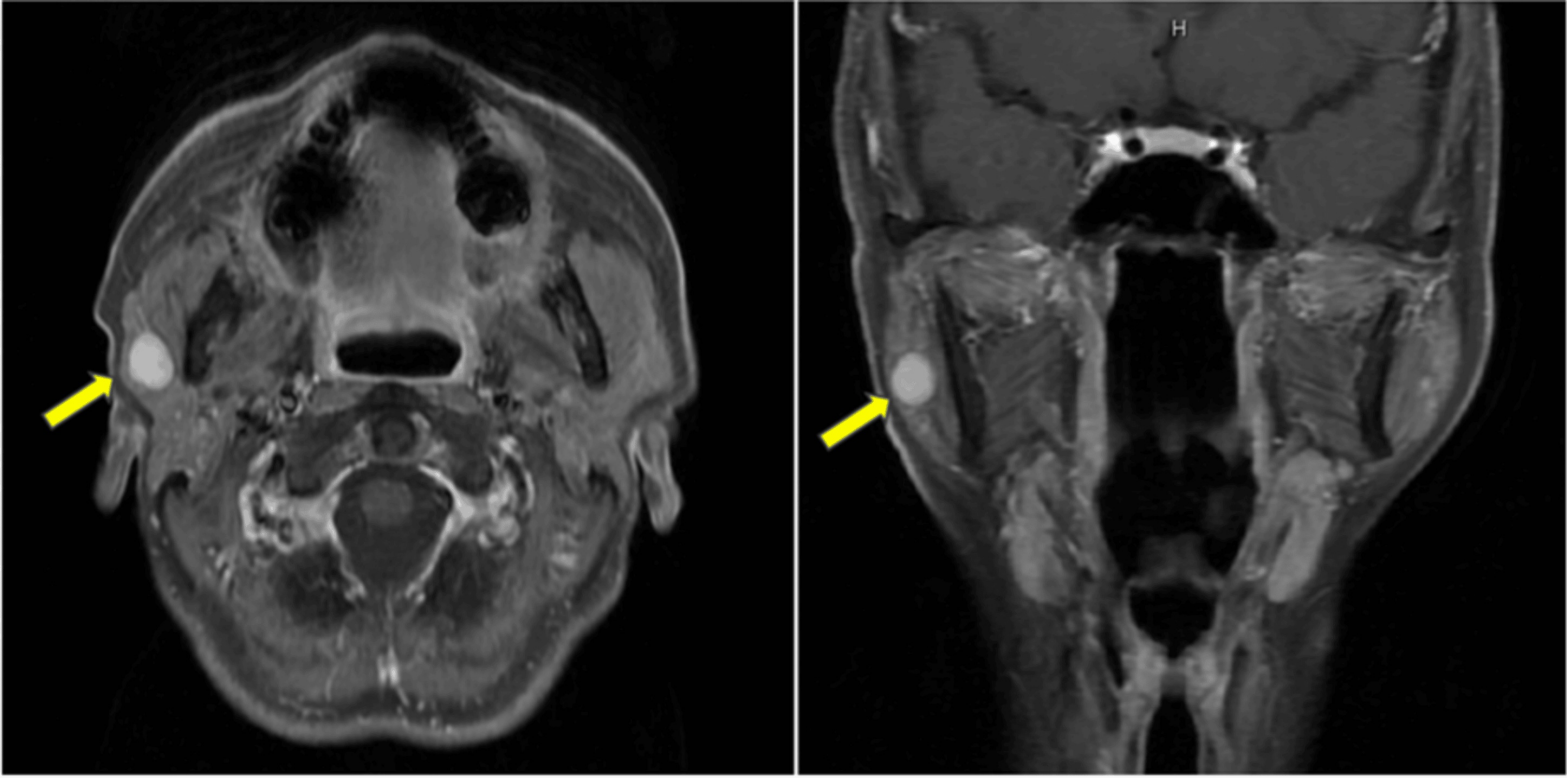
Preoperative T1-weighted gadolinium-enhanced MRI image The yellow arrow indicates the tumor. A mass with a well-defined margin measuring 10 × 14 × 12 mm and showing homogeneous contrast enhancement is noted in the right preauricular region.

A 50-year-old woman in good health was referred to our department complaining of a tumor in front of her right ear that had been present for over a year. She had no medical history other than a cesarean section and had never had shingles. The physical examination revealed a movable, elastic, hard mass measuring approximately 15 mm in front of the right ear, but no facial nerve palsy was observed. Laboratory tests revealed no abnormalities. MRI demonstrated a well-defined mass measuring 10 × 14 × 12 mm in the right parotid region, appearing T1-low, T2-high, and showing contrast enhancement (Figure 1). Fine needle aspiration cytology revealed atypical cells but no malignant findings. We proposed surgical resection for both diagnostic and therapeutic purposes and performed a parotid gland lobectomy four months after the initial consultation.

**Figure 2:**
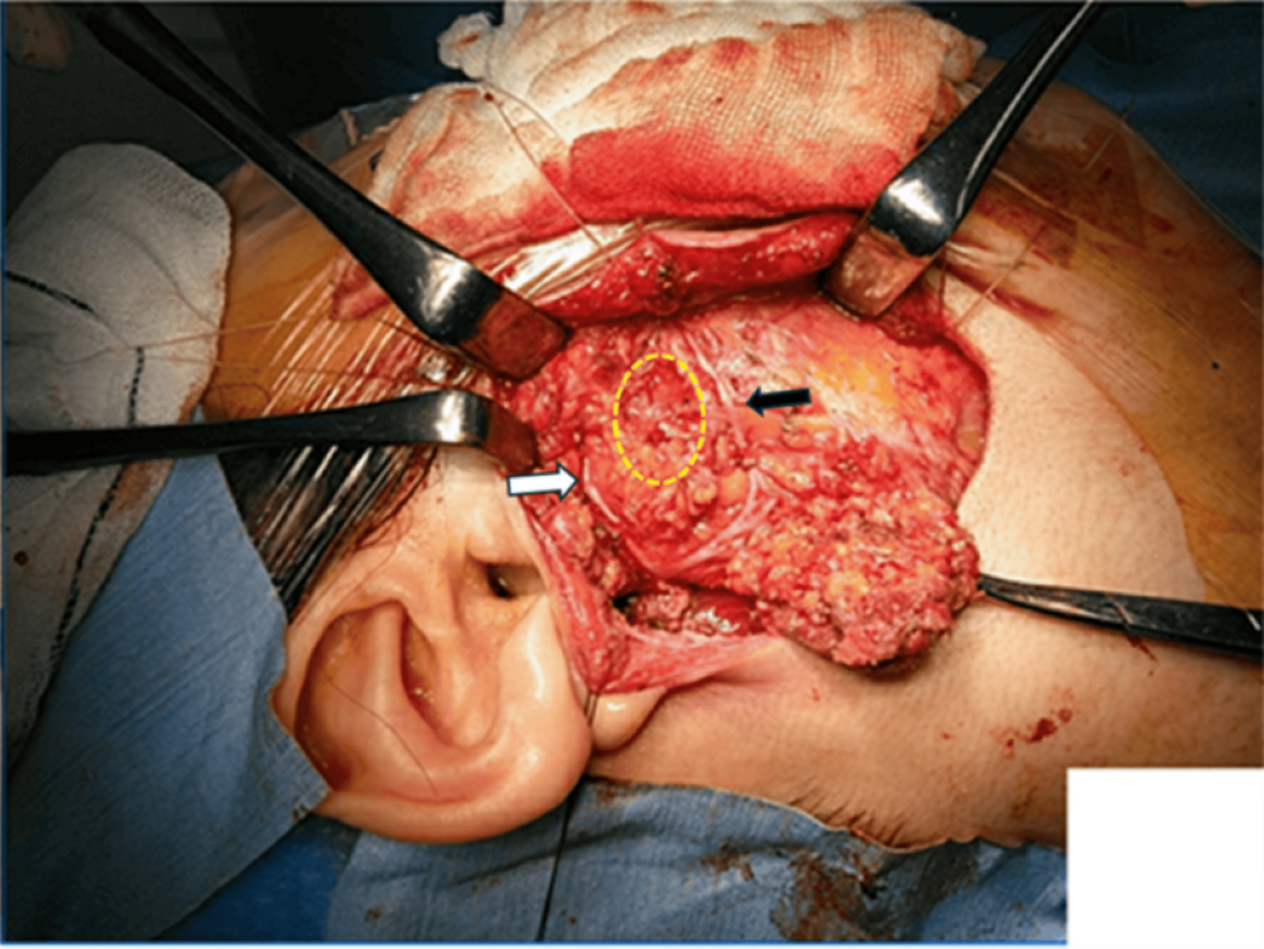
Intraoperative findings after tumor resection The left side of the image is cephalic, and the right side is caudal. The yellow dotted circle indicates the location where the tumor was present. The white arrow indicates the zygomatic branch of the facial nerve, and the black arrow indicates the buccal muscle branch.

Under general anesthesia, a standard S-shaped incision was made. The main trunk of the facial nerve was identified, and the ascending and descending branches were distinguished. From these, the zygomatic branch and the buccal muscle branch, each extending anteriorly and centrally, were identified. The tumor was located between the zygomatic branch and the buccal muscle branch, and both nerves were preserved during its removal. After tumor removal, the main trunk of the facial nerve was stimulated using a nerve stimulator, confirming that all facial muscles responded normally (Figure 2). To prevent Frey's syndrome, the parotid capsule was partially sutured, taking care to avoid tension.

Mild paralysis of the lower lip movement was observed postoperatively, but movement of the other facial muscles was normal. The House-Brackmann classification was Grade II. The postoperative course was uneventful, and the patient was discharged on the eighth postoperative day. Pathological findings confirmed a benign basal cell adenoma. Postoperative pain had improved during the hospital stay. However, she began feeling pain around her right ear on the 10th day after surgery. Within a few days, the pain intensified to her right temple, and she was taking pain medication. On postoperative day 16, the patient noticed right facial motor paralysis and sought emergency care. Severe motor paralysis was observed across the entire right side of the face, classified as Hause-Brackmann Grade V. No signs of infection such as redness or swelling, nor tenderness, were observed at the surgical site of the parotid gland. Pain was present from the right auricle to the temporal region, but no rash was observed. The patient was admitted the same day for further examination and treatment.

**Figure 3:**
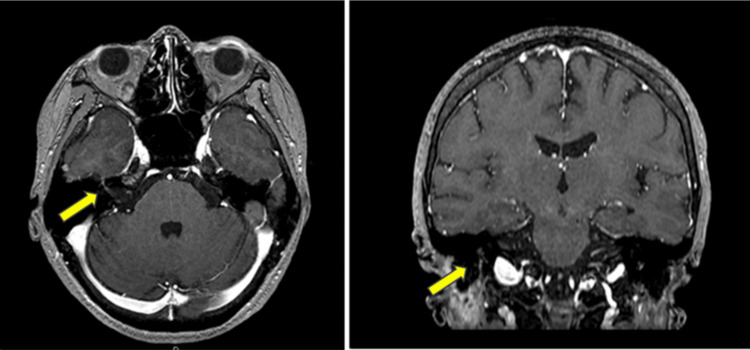
T1-weighted gadolinium-enhanced MRI image at the time of readmission The facial nerve running within the right temporal bone is enhanced compared to the left side (yellow arrow).

Laboratory tests showed no increase in white blood cells or elevated CRP levels (Table 1). MRI revealed only postoperative changes in the parotid region, with no evidence of abscess formation or tumor recurrence. Furthermore, contrast enhancement of the facial nerve was observed within the right temporal bone, indicating inflammatory edema within the facial nerve (Figure 3). Based on these findings, the possibility of delayed postoperative neurogenic edema or neuritis caused by viral reactivation, but a definitive diagnosis could not be established. While evaluating herpesvirus antibody titers, treatment with steroids and antiviral drugs for varicella-zoster virus (VZV) was initiated in accordance with the management of severe idiopathic facial nerve palsy. Prednisolone sodium succinate was administered intravenously starting at 100mg/day, then gradually tapered to 40mg/day over one week. Subsequently, oral prednisolone was administered at 30mg/day, gradually tapered to 5mg/day every other day over one week and then discontinued. Concurrently, acyclovir 5mg/kg every eight hours was administered via IV drip infusion for one week. Electroneurography (ENoG) was performed on the seventh day after onset. The amplitude of electrically evoked muscle action potential was 27% compared to the unaffected side.

**Table 1:** Patient’s laboratory data at the onset of delayed facial nerve palsy (DFNP)

Parameter	Value	Normal range
White blood cell count	5.1 × 10^3^/μl (high)	3.3–8.6 × 10^3^/μl
Hemoglobin	11.5 g/dl	11.6–14.8 g/dl
Platelets	207 × 10^3^/μl	158–348 × 10^3^/μl
Amylase	201 U/l	44–132 U/l
Sodium	141m Eq/l	138–145 mEq/l
Potassium	4.2 mEq/l	3.6–4.8 mEq/l
C-reactive protein	0.01 mg/dl	0.46–0.79 mg/dl
Creatinine	0.68 mg/dl	0.46–0.79 mg/dl

Table 2 shows antibody titers for herpes simplex virus (HSV) and VZV measured by enzyme immunoassay at the time of onset and one month after treatment. Both HSV-IgM antibodies and VZV-IgM antibodies were negative at the start of treatment. Although HSV-IgG antibody titers were negative after treatment, VZV-IgG antibody titers showed a slight increase. The patient was subsequently monitored as an outpatient, and the paralysis had completely resolved four months after onset.

**Table 2:** Serum antibody titers at onset of delayed facial nerve palsy (DFNP) and at completion of treatment HSV; herpes simplex virus, VZV; varicella-zoster virus

Enzyme immunoassay (unit: index)	Value		Biological reference range		
	Onset	After treatment	Negative	Equivocal	Positive
HSV-IgM	0.20	0.34	<0.80	0.80-1.20	1.21≤
HSV-IgG	<2.0	<2.0	<2.0	2.0-3.9	4.0≤
VZV-IgM	0.17	0.35	<0.80	0.80-1.20	1.21≤
VZV-IgG	8.6	12.9	<2.0	2.0-3.9	4.0≤

## Discussion

DFNP is defined as facial nerve palsy on the surgical side that develops more than 72 hours after surgery [2]. Although it is an extremely rare condition, it has been reported in otolaryngological surgeries involving the perineural canal or temporal bone [3]. Among these, the incidence of DFNP in acoustic neuroma surgery has been reported to be relatively high at 14.5-29.5% [4,5]. On the other hand, in ear surgery, procedures involving the temporal bone, such as tympanoplasty with mastoidectomy, stapes surgery, cochlear implant placement, and endolymphatic sac fenestration, have been reported to cause DFNP. Among these, the incidence of DFNP is 1.14-1.9% in tympanoplasty and 0.7-1.7% in cochlear implant surgery, and 0.2-0.5% in stapes surgery [6-10]. These reports suggest possible factors for delayed paralysis include postoperative inflammatory edema, impaired blood flow due to temporal bone hematoma formation, and neuritis caused by reactivation of VZV in the geniculate ganglion due to surgical stress [11]. DFNP occurring relatively early, within three to five days postoperatively, is thought to be caused by postoperative edema, whereas cases attributed to herpes reactivation are presumed to occur more belatedly [3]. Indeed, a report of DFNP occurring 14 days after middle ear surgery showed significant changes in VZV antibody titers around the time of onset, suggesting reactivation as the cause [12]. On the other hand, reports of DFNP occurring more than 72 hours after parotid gland surgery are extremely rare. Although the mechanism and frequency remain unclear, delayed edema and viral reactivation are presumed, similar to temporal bone surgery. Furthermore, it cannot be ruled out that idiopathic facial nerve palsy coincidentally developed on the surgical side at the postoperative timing. In this case, although the facial nerve itself is directly exposed, this area is not covered by bone, making the risk of ischemia due to nerve edema lower than within the temporal bone. While residual parotid glands may temporarily swell after parotid surgery, in this case, tension was avoided during parotid capsule suturing to prevent ischemia caused by edema. However, even after performing these procedures, it is difficult to rule out postoperative inflammatory edema or ischemia. On the other hand, the appearance of pain around the ear and in the temporal region, which was not present immediately after surgery, suggests the possibility of VZV reactivation.

Previous reports indicate that herpes zoster neuritis is associated with approximately 29% of idiopathic facial nerve palsies [13]. Furthermore, auricular pain and pain in the temporal region are strong indicators of facial nerve palsy caused by VZV [14]. Serological diagnosis based on VZV antibody titers requires either elevated IgM levels or a four-fold or greater increase in IgG levels between acute and convalescent phases [15]. Since the frequency of positive IgM antibodies is not high, diagnosis relies on IgG; however, IgG is not suitable for acute-phase diagnosis. Furthermore, an increase in IgG is not observed in all cases. Choi et al. reported that an increase in IgG antibody titers was observed in 72.7% of patients who developed VZV, but only 36.4% showed a fourfold or greater increase [16]. Although this case demonstrated a mild increase in IgG antibody titers, it was less than fourfold and therefore not significant. However, as mentioned above, it could not be ruled out that VZV reactivation contributed to the onset of pain from the auricle to the temporal region. On the other hand, relying solely on the presence of surrounding pain for diagnosis is dangerous. It is necessary to evaluate the presence of recurrence, infection, hematoma, or swelling that could cause pain through blood tests and imaging studies.

Steroid treatment is the most important therapy for DFNP. The recovery rate for DFNP is considered to be higher than that for immediate paralysis [4]. The case reported by Ostrowski et al. following parotid surgery presented with unilateral paralysis without pain starting on the 12th postoperative day and recovered well after oral steroid therapy [17]. In our case, treatment with antiviral medication and steroids was administered. The course was favorable, with improvement in paralysis and no recurrence observed for over a year. In this case, serological confirmation of VZV was not obtained, and the significance and efficacy of antiviral drugs remain unclear. Combination therapy in idiopathic facial nerve paralysis has been reported to reduce synkinesis, although it does not affect recovery rates [18]. In our country, it is weakly recommended for severe cases, and it may be considered in clinically suspected severe cases of viral reactivation.

## Conclusions

We encountered a case of delayed complete facial nerve palsy following benign parotid gland surgery. The palsy improved with combined treatment using steroids and antiviral medication. Although delayed paralysis after parotid gland surgery is extremely rare compared to middle ear/temporal bone surgery, if it occurs, the most important thing is to carefully investigate the several causative factors including postoperative infection, edema, hematoma, tumor recurrence, and viral reactivation.
